# Causative Classification of Ischemic Stroke by the Machine Learning Algorithm Random Forests

**DOI:** 10.3389/fnagi.2022.788637

**Published:** 2022-04-15

**Authors:** Jianan Wang, Xiaoxian Gong, Hongfang Chen, Wansi Zhong, Yi Chen, Ying Zhou, Wenhua Zhang, Yaode He, Min Lou

**Affiliations:** ^1^Department of Neurology, School of Medicine, The Second Affiliated Hospital of Zhejiang University, Hangzhou, China; ^2^Department of Neurology, Jinhua Hospital of Zhejiang University, Jinhua Municipal Central Hospital, Jinhua, China

**Keywords:** machine learning, cardioembolism, large-artery atherosclerosis, small-artery occlusion, stroke

## Abstract

**Background:**

Prognosis, recurrence rate, and secondary prevention strategies differ by different etiologies in acute ischemic stroke. However, identifying its cause is challenging.

**Objective:**

This study aimed to develop a model to identify the cause of stroke using machine learning (ML) methods and test its accuracy.

**Methods:**

We retrospectively reviewed the data of patients who had determined etiology defined by the Trial of ORG 10172 in Acute Stroke Treatment (TOAST) from CASE-II (NCT04487340) to train and evaluate six ML models, namely, Random Forests (RF), Logistic Regression (LR), Extreme Gradient Boosting (XGBoost), K-Nearest Neighbor (KNN), Ada Boosting, Gradient Boosting Machine (GBM), for the detection of cardioembolism (CE), large-artery atherosclerosis (LAA), and small-artery occlusion (SAO). Between October 2016 and April 2020, patients were enrolled consecutively for algorithm development (phase one). Between June 2020 and December 2020, patients were enrolled consecutively in a test set for algorithm test (phase two). Area under the curve (AUC), precision, recall, accuracy, and F1 score were calculated for the prediction model.

**Results:**

Finally, a total of 18,209 patients were enrolled in phase one, including 13,590 patients (i.e., 6,089 CE, 4,539 LAA, and 2,962 SAO) in the model, and a total of 3,688 patients were enrolled in phase two, including 3,070 patients (i.e., 1,103 CE, 1,269 LAA, and 698 SAO) in the model. Among the six models, the best models were RF, XGBoost, and GBM, and we chose the RF model as our final model. Based on the test set, the AUC values of the RF model to predict CE, LAA, and SAO were 0.981 (95%CI, 0.978–0.986), 0.919 (95%CI, 0.911–0.928), and 0.918 (95%CI, 0.908–0.927), respectively. The most important items to identify CE, LAA, and SAO were atrial fibrillation and degree of stenosis of intracranial arteries.

**Conclusion:**

The proposed RF model could be a useful diagnostic tool to help neurologists categorize etiologies of stroke.

**Clinical Trial Registration:**

[www.ClinicalTrials.gov], identifier [NCT01274117].

## Introduction

At present, stroke is one of the major global health problems, with 113 million disability-adjusted life years (DALYs) per year, while more than 80% of DALYs occur in low-income and middle-income countries (LMICs) (Pandian et al., 2018, 2020). The recurrent strokes, frequently ischemic and more disabling and costly than the first stroke, constitute a notable proportion (25–30%) of all preventable strokes (Hankey, 2014; Campbell and Khatri, 2020). Therefore, how to make an effective secondary prevention strategy is crucial to reduce the stroke burden.

Secondary prevention strategies of acute ischemic stroke (AIS) vary by different etiologies (Sacco et al., 1989b; Petty et al., 2000; Lovett et al., 2004). Currently, the most widely accepted ischemic stroke subtyping system is the Trial of ORG 10172 in acute stroke treatment (TOAST) classification scheme (Adams et al., 1993; Chen et al., 2012). However, even with extensive testing, prompt identification is challenging to physicians in clinical practice (Hankey, 2014; Jauch et al., 2017). Neurologists, who lack comprehensive knowledge of etiology categorization, especially junior neurologists, could not make an accurate assessment of etiology. Previous studies have reported that junior neurologists (less than 5 years of experience in clinical neurology) had a lower inter-observer reliability of etiological classification than senior neurologists (more than 12 years of practical experience) (κ = 0.36 vs. κ = 0.74), as junior neurologists were usually ambiguous about the medical record (Goldstein et al., 2001; Meschia et al., 2006; Yang et al., 2019). The inaccurate assessment of etiology definitely leads to poor secondary prevention of stroke, which would become more prominent in the countries with a large population but a less well-financed national health system (Pandian et al., 2020). Thus, alternative methods for rapid and accurate causative classification are needed to improve the secondary prevention strategies of AIS for junior neurologists, even for general practitioners.

Population-level studies from LMICs reported a high prevalence of stroke, and any future increases in stroke rates will most likely be driven by LMICs; people in LMICs more often had severe strokes (Pandian et al., 2020). Given the growing burden of stroke in LMICs, emphasizing strategies for reducing the risk of stroke in these regions was crucial to relieve the global stroke burden (Pandian et al., 2018). Furthermore, studies have proven that consistent secondary prevention was the key element to improve post-discharge morbidity and mortality, and mHealth, using mobile phones to provide patients and healthcare workers with support to improve health, had potential benefits in LMICs (Pandian et al., 2018, 2020). The excellent prediction performance with the applications of machine learning (ML) in many healthcare areas has inspired innovations in the development of novel ML-based stroke etiology diagnostic technology. ML models can capture complex, nonlinear relationships in medical data and learn the features to classify the entity they describe, which may be appropriate to mimic the process of etiology assessment. Therefore, in this study, we investigated whether the model using ML methods could precisely identify the cause of ischemic stroke by learning the features of the etiologies and tested its accuracy.

## Materials and Methods

### Study Setting

This study was a retrospective analysis based on a multicenter prospective registry, Computer-based Online Database of Acute Stroke Patients for Stroke Management Quality Evaluation (CASE-II, NCT04487340). Initiated in 2016, CASE-II was designed to examine the current status of stroke care in China, and the data would be used to help develop strategies to improve stroke care. All data were preserved in a safe information database and monitored by an independent contract research organization. We collected and analyzed all consecutive patients diagnosed with AIS within 24 h of onset with determined stroke subtypes. Patients with incomplete information for analysis (missing any data in data for developing models in Supplementary Material) were excluded. We excluded patients with stroke of undetermined cause (SUE) and stroke of other determined etiology (SOE) in the process of ML because the aim of this study was to identify three main causes of ischemic stroke.

The study was conducted in two phases. In phase one, eligible patients from 63 sites were enrolled and partitioned randomly into training (80%) and validation (20%) sets for algorithm development from October 2016 to April 2020. In phase two, eligible patients from 63 sites were enrolled in a test set from June 2020 to December 2020. The 63 sites from the test set were the same original 63 sites from the training and validation sets in phase one. In addition, there were no patients present in both phase 1 and phase 2. The models were developed through the learning of the etiological features in the training set, while validation set and test set were used to evaluate the model.

CASE-II (NCT04487340) was approved by the Human Ethics Committee of the Second Affiliated Hospital of Zhejiang University, School of Medicine, and the approval number is yan-2018-102. Written informed consent was obtained from each patient or an appropriate family member. Clinical investigation had been conducted according to the principles expressed in the Declaration of Helsinki.

### Data Information

The data collected and analyzed in the study included demographics; clinical information, such as baseline modified Rankin Scale (mRS) score, baseline National Institutes of Health Stroke Scale (NIHSS) score, history of hypertension, atrial fibrillation (AF), diabetes mellitus, hyperlipidemia, hyperhomocysteinemia, history of stroke/transient ischemic attack (TIA), smoking, alcohol drinking, body mass index (BMI), and vital signs; and laboratory tests and radiologic data during hospitalization (data for developing models in Supplementary Material).

### Assessment of Stroke Cause

Each patient’s stroke subtype was assessed by two experienced senior neurologists (more than 10 years of clinic practical experience and research experience in stroke), who were blinded to the study design and independently reviewed each patient’s available data, with any disputes settled *via* reviewing by a third experienced senior neurologist for a consensus decision. Etiological categories were defined by the TOAST criteria. Cardioembolism (CE) includes patients with arterial occlusions presumably due to an embolus arising in the heart. Large-artery atherosclerosis (LAA) includes patients with >50% atherosclerotic stenosis or atherosclerotic occlusion at the bifurcation of the carotid artery on the symptomatic side. Small-artery occlusion (SAO) includes patients who have one of the traditional clinical lacunar syndromes, have no evidence of cerebral cortical dysfunction, and have a normal computed tomography (CT)/ magnetic resonance imaging (MRI) examination or a relevant brain stem or subcortical hemispheric lesion with a diameter of less than 1.5 cm. Meanwhile, potential cardiac sources for embolism should be absent, and evaluation of the large extracranial arteries should not demonstrate stenosis of greater than 50% in an ipsilateral artery. SUE includes the following: (1) ≥2 potential causes of stroke, or (2) no likely etiology determined despite an extensive evaluation, or (3) no cause found due to inadequate evaluation. SOE includes patients with other determined pathogenesis subtypes, such as non-atherosclerotic vasculopathies, hypercoagulable states, or hematologic disorders. Diagnostic studies, such as blood tests or arteriography, should reveal one of these unusual causes of stroke. Cardiac sources of embolism and large-artery atherosclerosis should be excluded by other studies (Adams et al., 1993).

### Feature Selection

According to published literature and pathophysiological consideration, we first selected clinical information, including baseline mRS score, baseline NIHSS score, history of hypertension, AF, diabetes mellitus, hyperlipidemia, hyperhomocysteinemia, history of stroke/TIA, smoking, alcohol drinking, BMI, and vital signs, and laboratory tests and radiologic data during hospitalization (data for developing models in Supplementary Material; Hankey, 2014; Yan et al., 2017; Pandian et al., 2018; Campbell and Khatri, 2020). The feature selection was done exclusively with training set data. Then, we performed the score function based on Python packages Scikit-learn to score every factor using 5 methods, f_classif, mutual_info_classif, chi2, f_regression, mutual_info_regression, respectively. Based on the score, we chose the top 20 as candidate variables. Variables of five feature selectors selected for CE, LAA, and SAO models were the same. The 20 variables selected for the CE model were as follows: history of anticoagulants, previous coronary heart disease, previous atrial fibrillation, previous valvular heart disease, anterior circulation infarction, posterior circulation infarction, newly diagnosed atrial fibrillation, discharged atrial fibrillation, discharged valvular heart disease, discharged coronary heart disease, discharged heart failure, discharged hyperlipidemia, discharged diabetes mellitus, systolic blood pressure at admission, Baseline National Institutes of Health Stroke Scale score, platelet count, D-dimer, total cholesterol, triglyceride, and low-density lipoprotein. The 20 variables selected for the LAA model were as follows: female, history of anticoagulants, history of hypoglycemic drugs, alcohol drinking, previous coronary heart disease, previous atrial fibrillation, previous valvular heart disease, previous diabetes, anterior circulation infarction, posterior circulation infarction, large vessel occlusion, newly diagnosed atrial fibrillation, discharged atrial fibrillation, discharged valvular heart disease, discharged coronary heart disease, discharged heart failure, discharged diabetes mellitus, discharged deep venous thrombosis, degree of stenosis of intracranial arteries, and triglyceride. The 20 variables selected for the SAO model were as follows: history of anticoagulants, previous coronary heart disease, previous atrial fibrillation, posterior circulation infarction, large vessel occlusion, newly diagnosed atrial fibrillation, discharged atrial fibrillation, discharged coronary heart disease, systolic blood pressure at admission, diastolic blood pressure at admission, body mass index, Baseline Glasgow Coma Scale score, degree of stenosis of intracranial arteries, hemoglobin, platelet count, D-dimer, glucose at admission, total cholesterol, triglyceride, and low-density lipoprotein. Then, the further important factor selection and the redundant factor exclusion were performed using the Scikit-Learn package in Python software in the light of whether there was a correlation between the factor and the cause. There remained 6 variables in the CE model, 16 variables in the LAA model, and 15 variables in the SAO model after these two steps.

### Data Analysis

We developed and validated three multi-class algorithms and six common binary classification algorithms using the Scikit-Learn package in Python software (Pedregosa et al., 2011; Venthur et al., 2015). The highest area under the curves (AUCs) of the multi-class algorithms were lower than binary classification algorithms [0.962 (95%CI, 0.951–0.970) vs. 0.981 (95%CI, 0.978–0.986) for CE; 0.903 (95%CI, 0.897–0.911) vs. 0.919 (95%CI, 0.911–0.928) for LAA; and 0.909 (95%CI, 0.900–0.916) vs. 0.918 (95%CI, 0.908–0.927) for SAO], potentially due to the imbalance classifier nature of the multi-class algorithm. Thus, we chose the binary classification algorithms as the final algorithms. Details for the performance of the binary classification algorithms were as follows:

Random Forests (RF), Logistic Regression (LR), Extreme Gradient Boosting (XGBoost), K-Nearest Neighbor (KNN), Ada Boosting, and Gradient Boosting Machine (GBM) were selected. The data of phase one were randomly stratified (8:2) to the training and validation set for developing models, and the data of phase two were used as the test set for evaluating the models’ performance. The models were then developed using the retained features. During the preprocessing of the dataset, we used “value-min/(max-min)” to normalize the variables. Procedural details on how the Scikit-Learn package selects predictors can be found in papers published by Abraham et al. (2014). Five-fold cross-validation was used for the model derivation and internal evaluation by dividing the training set into five mutually exclusive parts, four of which were used as training data for the model derivation and one for evaluation as inner validation data; this process was repeated five times to generate five different but overlapping training data and five unique validation data.

In the training step, we optimized model hyperparameters of RF, LR, KNN, and Ada Boosting with a grid search algorithm (plots of hyperparameters search in Supplementary Material) and adopted the default value of hyperparameters of GBM and XBGoost. Each model’s hyperparameters and values were as follows: Hyperparameters of KNN are n_neighbors, leaf_size, weights, and algorithm, respectively, and values of n_neighbors are 3, 5, 8, 10, 15; values of leaf_size are 3, 5, 10; values of weights are uniform, distance; values of algorithm are auto, ball_tree, kd_tree, brute.

Hyperparameters of RF are n_estimators, max_depth, bootstrap, max_features, min_samples_leaf, and min_samples_split, respectively, and values of n_estimators are 30, 50; values of max_depth are 5, 10, 15, 20; values of bootstrap are True, False; values of max_features are 5, 8, None; values of min_samples_leaf are 5, 10; values of min_samples_split are 5, 10.

Hyperparameters of LR are C, penalty, max_iter, respectively, and values of C are 0.001, 0.01, 0.1, 1; values of penalty are l1, l2; values of max_iter are 150, 300. Hyperparameters of Ada Boosting are n_estimators, and learning_rate, respectively, and values of n_estimators are 10, 30, 50; values of learning_rate are 1, 0.5. Hyperparameters of XGBoost are learning_rate, n_estimators, and max_depth, respectively, and values of learning_rate are 0.1, 0.5, 1; values of n_estimators are 10, 25, 50; values of max_depth are 5, 10, 15.

Hyperparameters of GBM are learning_rate, n_estimators, and max_depth, respectively, and values of learning_rate are 0.1, 0.5, 1; values of n_estimators are 10, 25, 50; values of max_depth are 1, 5.

During the searching process, we set the AUC of receiver operating characteristic (ROC) as the score. To assess the generalizability of each model, we evaluated the predictive performance of all models on the test set. After determining the best model on the test set to predict CE, LAA, and SAO, respectively, we combined these three models into one new model for predicting a certain patient who belongs to one of the three causes. The algorithm to determine the certain cause was based on the probability of the three causes, and the final cause was the one that has the highest probability.

### Definitions of Metrics

To measure the performance of the classifiers, we use the conventional definitions of recall, precision, F1 score, and accuracy. In the descriptions below, we used the abbreviations TP (true positive), TN (true negative), FP (false positive), and FN (false negative) to describe correct and incorrect assignments of an unknown etiology to a predicted type, as described by this confusion matrix:

Recall: The proportion of samples of a particular stroke subtype that are correctly assigned to that type:


Recall=TP/(TP+FN).

Precision: The proportion of samples assigned to a particular type that are truly that type:


Precision=TP/(TP+FP).

F1 score: The harmonic mean of recall and precision:


F1=2⁢(recall*precision)/(recall+precision).

Accuracy: The ratio of the number of samples correctly classified by the classifier to the total number of samples:


Accuracy=(TP+TN)/(TP+TN+FP+FN)

Gini importance is a measurement of the feature importance: The importance of a feature is computed as the total reduction of the criterion brought by that feature.

### Code and Data Availability

The code and data used to generate results shown in this study are available from the author M.L. upon request.

### Statistical Analysis

Clinical characteristics were summarized by computing the median (interquartile range), and differences between two groups were estimated by the *t*-test or the Mann-Whitney *U*-test if they were continuous variables. Categorical or binary datum was summarized by proportion (n); differences between two groups were estimated by the Pearson χ^2^-test. ROC analysis was used to get the AUC of the prediction models. The ROC-derived optimal cutoff was determined at the maximal Youden Index. All statistical analysis was performed using SPSS, Version 22.0 (IBM, Armonk, New York). All comparisons were two-sided, with statistical significance defined as *P* < 0.05.

## Results

### Study Population

Between October 2016 and April 2020, a total of 18,209 patients were included in phase one, and from June 2020 to December 2020, a total of 3,688 patients were finally included in phase two. Of the included patients in phase one vs. phase two, mean age was 71 (62–80) vs. 70 (60–79) years, the number of female patients were 7,488 (41.1%) vs. 1,442 (39.1%), and median NIHSS on admission was 3 (1–7) vs. 3 (1–6). In phase one, the proportion of CE subtype was the highest (6,089, 33.4%), followed by LAA (4,539, 24.9%), SUE (4,481, 24.6%), SAO (2,962, 16.3%), and SOE (138, 0.8%). ML models enrolled all patients with CE, LAA, and SAO, and thus, 13,590 patients were included, with 10,872 cases (80%) in the training set (4,871 CE cases; 3,631 LAA cases; 2,370 SAO cases), and 2,718 cases (20%) in the validation set (1,218 CE cases; 908 LAA cases; 592 SAO cases). In phase two, the proportion of LAA subtype was the highest (1,269, 34.4%), followed by CE (1,103, 29.9%), SAO (698, 18.9%), SUE (597, 16.2%), and SOE (21, 0.6%); 3,070 patients (1,103 CE cases; 1,269 LAA cases; 698 SAO cases) were involved in the models. The baseline characteristics of patients included in the cohorts of algorithm development and test were listed in Table 1.

**TABLE 1:** Comparison of clinical characteristics between a cohort of algorithm development and a cohort of algorithm test.

	Cohort of algorithm development (*n* = 13,590)	Cohort of algorithm test (*n* = 3,070)	*P*-value
Female, *n* (%)	5688 (41.8)	1214 (39.5)	0.019
Age, year, median (IQR)	72 (63–80)	70 (61–80)	<0.001
Baseline mRS score, median (IQR)	2 (1–4)	2 (1–4)	<0.001
Baseline NIHSS score, median (IQR)	3 (1–8)	3 (1–6)	<0.001
GCS score, median (IQR)	15 (13–15)	15 (14–15)	<0.001
SBP at admission, mmHg, median (IQR)	151 (135–167)	151 (134–166)	0.523
DBP at admission, mmHg, median (IQR)	84 (75–94)	84 (75–94)	0.859
Glucose at admission, mmol/L, median (IQR)	5.3 (4.7–6.5)	5.3 (4.7–6.4)	0.018
BMI, kg/m^2^, median (IQR)	23.2 (20.9–25.6)	23.5 (21.5–25.7)	0.001
Hypertension, *n* (%)	8841 (65.1)	1955 (63.7)	0.135
Diabetes mellitus, *n* (%)	2673 (19.7)	559 (18.2)	0.065
Atrial fibrillation, *n* (%)	2941 (21.6)	507 (16.5)	<0.001
Hyperlipemia, *n* (%)	218 (1.6)	42 (1.4)	0.341
Smoking, *n* (%)	4379 (32.2)	1071 (34.9)	0.004
Alcohol drinking, *n* (%)	4766 (26.2)	966 (26.2)	0.682
Coronary heart disease, *n* (%)	1070 (7.9)	178 (5.8)	<0.001
Myocardial infarction, *n* (%)	113 (0.8)	22 (0.7)	0.521
Valvular heart disease, *n* (%)	296 (2.2)	49 (1.6)	0.041
Mitral stenosis, *n* (%)	99 (0.7)	23 (0.7)	0.903
Hyperhomocysteinemia, *n* (%)	20 (0.1)	5 (0.2)	0.839
Previous transient ischemic attack, *n* (%)	57 (0.4)	8 (0.3)	0.202
History of stroke, *n* (%)	2962 (21.8)	635 (20.7)	0.176
Renal insufficiency, *n* (%)	206 (1.5)	47 (1.5)	0.951
CE, *n* (%)	6089 (44.8)	1103 (35.9)	<0.001
LAA, *n* (%)	4539 (33.4)	1269 (41.3)	<0.001
SAO, *n* (%)	2962 (21.8)	698 (22.7)	0.256

*BMI, body mass index; CE, cardioembolism; DBP, diastolic blood pressure; GCS, Glasgow Coma Scale; IQR, interquartile range; LAA, large-artery atherosclerosis; mRS, modified Rankin Scale; NIHSS, National Institutes of Health Stroke Scale; SAO, small-artery occlusion; SBP, systolic blood pressure.*

The distributions of patients in phase one and phase two were not the same. Compared with patients in phase one, those in phase two were less likely to be female patients, have AF, have coronary heart disease, have CE and SUE, and more likely to be younger, have smoking habit, have LAA and SAO, and present lower baseline mRS and NIHSS score, have glucose at admission, and have higher Glasgow Coma Scale score and BMI at admission (Table 1).

### Feature Selection and Importance of Features Contributing to the Identification of Cardioembolism, Large-Artery Atherosclerosis, and Small-Artery Occlusion

The final CE model incorporated 6 variables, including discharged AF, previous AF, newly diagnosed AF, posterior circulation infarction, anterior circulation infarction, baseline NIHSS score, and age. The final LAA model included 16 variables, which were degree of stenosis of intracranial arteries, discharged AF, previous AF, newly diagnosed AF, large vessel occlusion, anterior circulation infarction, baseline mRS score, international normalized ratio, baseline NIHSS score, triglyceride, high-density lipoprotein, low-density lipoprotein, total cholesterol, proportion of neutrophils, and age. The 15 variables of the SAO model were stenosis of intracranial arteries, discharged AF, previous AF, baseline NIHSS score, newly diagnosed AF, large vessel occlusion, posterior circulation infarction, baseline mRS score, age, baseline GCS score, platelet count, triglyceride, international normalized ratio, proportion of neutrophils, and total cholesterol.

**FIGURE 1:**
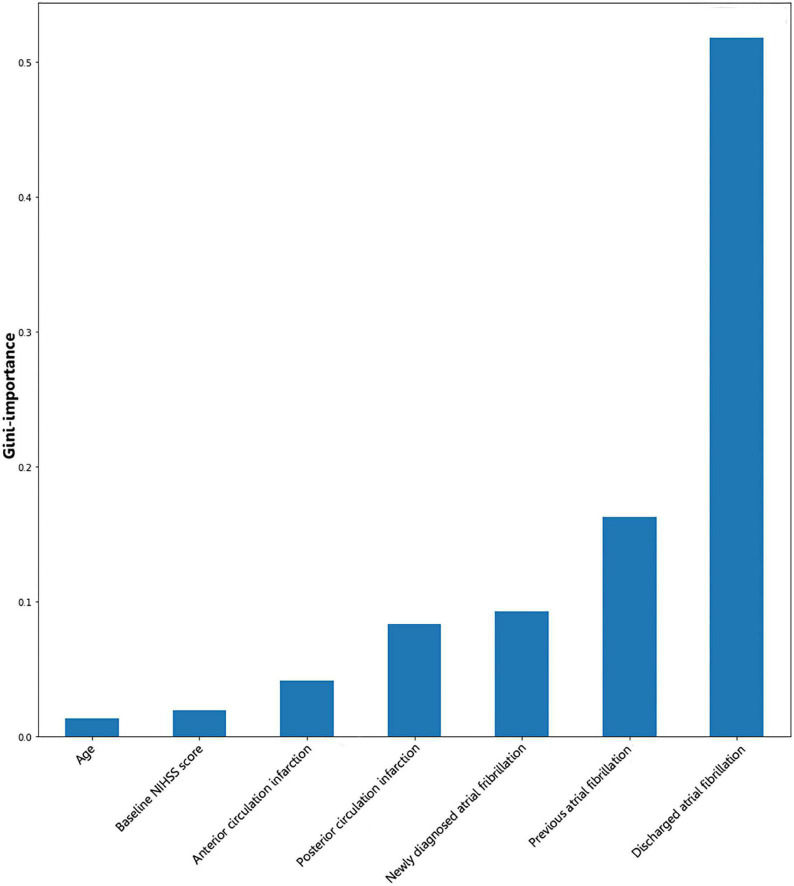
Illustration of features contributing to the identification of CE by Gini importance values. CE, cardioembolism; NIHSS, National Institutes of Health Stroke Scale. Gini importance is a measurement of the feature importance in the model; the higher the value of Gini importance is, the more important the feature is.

**FIGURE 2:**
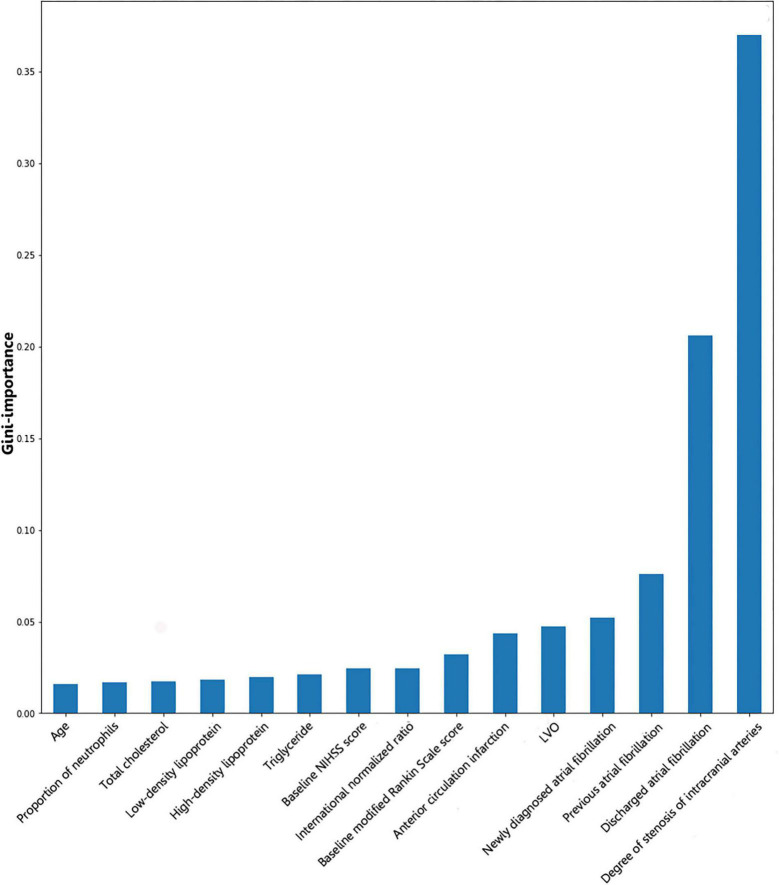
Illustration of features contributing to the identification of LAA by Gini importance values. LAA, large-artery atherosclerosis; LVO, large vessel occlusion; NIHSS, National Institutes of Health Stroke Scale. Gini importance is a measurement of the feature importance in the model; the higher the value of Gini importance is, the more important the feature is.

**FIGURE 3:**
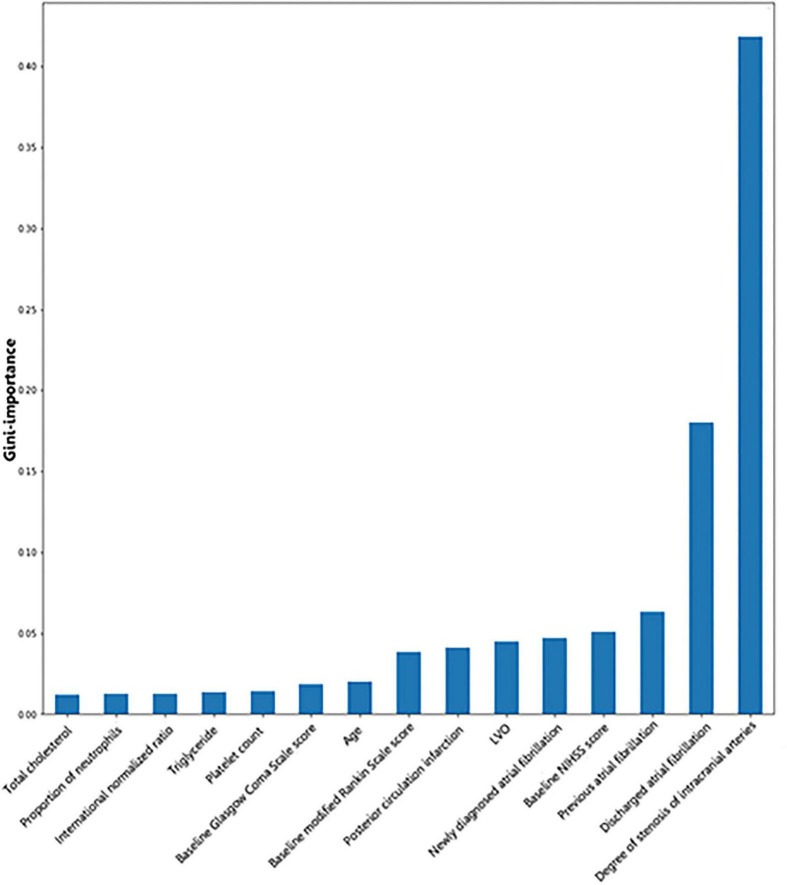
Illustration of features contributing to the identification of SAO by Gini importance values. LVO, large vessel occlusion; NIHSS, National Institutes of Health Stroke Scale; SAO, small-artery occlusion. Gini importance is a measurement of the feature importance in the model; the higher the value of Gini importance is, the more important the feature is.

Gini importance of every risk factor of CE, LAA, and SAO was calculated. As expected, AF was identified as the most significant contributor in the CE estimation, and other important items included posterior circulation infarction and anterior circulation infarction (Figure 1). The most significant items contributing to identifying LAA were stenosis degree of intracranial arteries, AF, and large vessel occlusion (Figure 2). In addition, the most important items of SAO classification were stenosis degree of intracranial arteries, AF, and baseline NIHSS score (Figure 3). Furthermore, we noted that some items easy to ignore in ordinary clinical practice (e.g., baseline NIHSS score, baseline mRS score, and age) also had some contributions to causative classification.

### Model Performance on the Test Set

The AUC, precision, recall, F1 score, and accuracy of each model on the test set were presented in Table 2. Among six models, the models with the best predictive performance were RF, XGBoost, and GBM, and there was no difference in AUC between these three models. The AUC of the best models to predict CE was as follows: RF, 0.981 (95%CI, 0.978–0.986); XGBoost, 0.982 (95%CI, 0.978–0.986); and GBM, 0.982 (95%CI, 0.979–0.987). The AUC of the best three models to predict LAA was as follows: RF, 0.919 (95%CI, 0.911–0.928); XGBoost, 0.920 (95%CI, 0.912–0.929); and GBM, 0.920 (95%CI, 0.978–0.986). The AUC values of these models to predict SAO were as follows: RF, 0.918 (95%CI, 0.908–0.927); XGBoost, 0.919 (95%CI, 0.910–0.928); and GBM, 0.919 (95%CI, 0.910–0.928). Finally, we chose the RF model as the final prediction model, because RF is a widely applied algorithm of being trained quickly and providing insights into the features that can predict the stroke etiology and has shown good predictive performance in the stroke field and other medical studies (Denisko and Hoffman, 2018; Jurmeister et al., 2019; Lee et al., 2020; Wang et al., 2020).

**TABLE 2:** Comparison of six models to predict etiology.

Etiology	AUC (95% CI)	Precision	Recall	F1 score	Accuracy
**CE**					
RF	0.981 (0.978–0.986)	0.955	0.955	0.955	0.958
LR	0.976 (0.971–0.981)	0.937	0.934	0.933	0.934
XGBoost	0.982 (0.978–0.986)	0.959	0.959	0.959	0.959
KNN	0.974 (0.970–0.980)	0.955	0.955	0.955	0.955
Ada Boosting	0.976 (0.971–0.981)	0.940	0.937	0.937	0.937
GBM	0.982 (0.979–0.987)	0.958	0.958	0.958	0.958
**LAA**					
RF	0.919 (0.911–0.928)	0.847	0.849	0.848	0.849
LR	0.866 (0.857–0.877)	0.785	0.771	0.775	0.771
XGBoost	0.920 (0.912–0.929)	0.846	0.848	0.846	0.848
KNN	0.902 (0.893–0.912)	0.833	0.836	0.833	0.836
Ada Boosting	0.916 (0.908–0.925)	0.845	0.847	0.845	0.847
GBM	0.920 (0.978–0.986)	0.846	0.848	0.846	0.848
**SAO**					
RF	0.918 (0.908–0.927)	0.864	0.864	0.864	0.864
LR	0.855 (0.843–0.868)	0.761	0.791	0.758	0.791
XGBoost	0.919 (0.910–0.928)	0.868	0.867	0.867	0.867
KNN	0.837 (0.824–0.851)	0.765	0.781	0.771	0.781
Ada Boosting	0.918 (0.909–0.927)	0.857	0.860	0.858	0.861
GBM	0.919 (0.910–0.928)	0.863	0.863	0.863	0.863

*AUC, area under the curve; CE, cardioembolism; CI, confidence interval; GBM, gradient boosting machine; KNN, K-nearest neighbor; LAA, large-artery atherosclerosis; LR, logistic regression; RF, random forests; SAO, small-artery occlusion; XGBoost, extreme gradient boosting. The model method is more effective when the F1 score is higher.*

**FIGURE 4:**
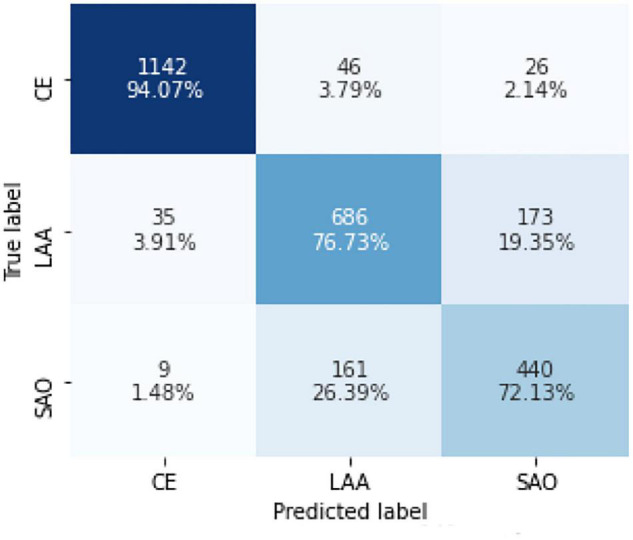
Confusion matrix of the model in identifying CE, LAA, and SAO on the test set. CE, cardioembolism; LAA, large-artery atherosclerosis; SAO, small-artery occlusion. Confusion matrices are calculated by comparing the position and classification of each measured sample with the actual corresponding position and classification. Each column represents the predicted category of the data, and each row represents the true attribution category.

Further analysis of the new model that combined the three best models showed 1,142 (94.07%) CE cases, 686 (76.73%) LAA cases, and 440 (72.13%) SAO cases, which were predicted correctly. Among the CE patients, 46 (3.79%) cases were incorrectly identified as LAA and 26 (2.14%) cases were incorrectly identified as SAO. Among the LAA patients, 35 (3.19%) cases were predicted to be CE and 173 (19.35%) cases were predicted to be SAO. Among the SAO patients, the prediction of 9 (1.48%) patients was CE, and the prediction of 161 (26.39%) patients was LAA. The accuracy of predicting LAA and SAO was lower than CE (Figure 4).

## Discussion

To our knowledge, it was the first study to implement ML to determine stroke etiologies based on large cohorts of algorithm development and test by far. Results showed that our proposed model categorized the stroke subtypes with excellent accuracy through integrating clinical information with radiologic data and laboratory testing. In addition, we also found the most important items for identifying stroke etiologies involved in heart and image information, including AF and stenosis degree of intracranial arteries.

Previous studies have put attention on CE. Imaging findings revealed that delayed-contrast filling sign (Zhou et al., 2019) and overestimation ratio of susceptibility vessel sign (Zhang et al., 2017) could predict CE, and the AUCs were 0.80 and 0.928, respectively. Compared with previous studies, our model showed better performance ability (0.981 vs. 0.80 and 0.928). There were several potential explanations for these findings. First, clinical information, including primary vital signs and the physical examination of patients with ischemic stroke, was important when determining ischemic stroke etiology (Garcia-Cazares et al., 2020). Our model enrolled the necessary data and captured nonlinear relationships, including interactions among the input parameters and outputs until reaching high accuracy (Koo et al., 2013), which might improve the predictive performance of the model. Second, the popularization of advanced diagnostic technology, including long-term monitoring to document paroxysmal AF and computed tomographic angiography, to visualize vessel pathologies (i.e., plaque, degree of stenosis), could be useful in reducing the proportion of cryptogenic stroke and revealing the underlying mechanisms of the index stroke.

Interestingly, we found that AF and stenosis degree of intracranial arteries were the most important items for determining CE, LAA, and SAO. This is consistent with previous studies which reported that AF was the common cause of CE, and stenosis degree of intracranial arteries was the most important component to diagnose LAA (Adams et al., 1993; Hankey, 2014; Boodt et al., 2020). According to TOAST criteria, potential large-artery atherosclerotic sources of thrombosis or embolism should be eliminated when CE was diagnosed, and the diagnosis of LAA should exclude potential sources of CE; meanwhile, potential cardiac sources for embolism and stenosis of greater than 50% in an ipsilateral artery should not be revealed when diagnosing SAO (Adams et al., 1993). Thus, these features were closely related to etiological assessment, and the sensitivity and specificity of the model could be significantly improved when these items were incorporated.

Notably, the characteristics of patients in the test group were different from those in the model-developed group, while our model showed excellent performance ability in both groups, suggesting the generalizability of the algorithm. There are some potential application scenarios for our algorithm. Currently, the incidence of cryptogenic stroke is 25–39% in different registries (Sacco et al., 1989a; Petty et al., 2000; White et al., 2005). Previous studies have reported that an observer’s experience may affect the reliability of etiological classification systems. For example, junior neurologists had a lower inter-observer reliability value of TOAST than that of the senior neurologists (κ = 0.36 vs. κ = 0.74) (Goldstein et al., 2001; Meschia et al., 2006; Yang et al., 2019). According to the previous report, the overall accuracy of junior physicians judging the TOAST etiologies varied from 0.354 to 0.643, with the senior neurologists as the reference. The detailed accuracy of junior physicians judging LAA varied from 0.750 to 0.774, CE from 0.387 to 0.516, and SAO from 0.373 to 0.667, respectively (Goldstein et al., 2001; Selvarajah et al., 2009; Yang et al., 2019; Suo et al., 2020). Our model outperformed the junior neurologists (CE: 0.958; LAA: 0.849; SAO: 0.864). In this respect, our proposed model could provide a better tool for physicians at different levels to give a causative classification of ischemic stroke and even provide clues to neurologists when the diagnoses were disputed.

The inferior accessibility to stroke care services and clinical management, the lack of consciousness of prevention, the poor control of risk factors, and poor quality of diagnoses and treatments in LMICs may jointly lead to the great stroke burden in these regions (Yan et al., 2017; Pandian et al., 2018). In addition, patients with stroke in LMICs were usually treated by internists or family physicians, rather than by a stroke specialist, because stroke units and trained specialists in stroke care were scarce in LMICs (Pandian et al., 2020). Therefore, strategies to help improve the knowledge and awareness of stroke of physicians in LMICs were very crucial for reducing the stroke burden in these regions. Furthermore, because of the growing ownership of mobile phones worldwide, there was great potential to use mHealth in LMICs, particularly in regions with poor access to healthcare (Pandian et al., 2018). Our proposed model with excellent accuracy could enable patient-centered messaging and precisely provide the clues of stroke causes, which was effective in the secondary prevention of stroke. In addition, our model provided the most important items to identify stroke causes, which could help improve the knowledge and awareness of stroke in physicians and help them to better treat and manage the stroke in the future.

We noted that there were other etiological stroke classification systems besides TOAST (such as Causative Classification System, Atherosclerosis, Small-Vessel Disease, Cardiac Source, Other Cause, Chinese Ischemic Stroke Subclassification, magnetic resonance imaging-based diagnostic algorithm for AIS subtype classification, Stop Stroke Study TOAST), which try to improve upon the TOAST system (Ay et al., 2005; Chen et al., 2012; Ko et al., 2014). Stroke causes can be complex and multifactorial. Our proposed model is just the beginning to make primary assessments for stroke etiologies, provides clues that are most relevant with stroke for physicians, and does not aim to stop the workup when one etiology is identified. In addition, clinicians should consider other stroke-relevant risk factors on the basis of the assessments of our model to further find the exact cause and comprehensively understand the disease in clinical practice. In this way, physicians could comprehensively evaluate the conditions and further perform the optimal prevention measures.

## Limitations

Limitations of this study should be noted. First, the number of input parameters can be large, and the use of the model would require integration with communication systems and clinical database systems or other image storage databases, however, which is relatively easy to achieve in modern hospital systems. The model could be implemented as a rapid diagnostic tool to flag patients with different etiologies when radiologic data and clinical information are available, and neurologists could review these suspected cases identified by the model with a higher priority. In addition, we found two variables in LAA and SAO models, namely, degree of stenosis of intracranial arteries and large vessel occlusion, which might not be available in LMICs. But our proposed model still had a good prediction power when we tried to run the model in the absence of these two variables. In the absence of these two variables, the AUCs of LAA and SAO were 0.756 (95%CI, 0.744–0.766) and 0.794 (95%CI, 0.788–0.803). Actually, we still recommended the popularization of vascular examination, such as CTA, for better management of stroke patients. Second, the accuracy of identifying LAA and SAO was low, and we should take further study to improve the prediction of LAA and SAO. Third, this algorithm was developed using a cohort of only Chinese stroke patients. There was some potential limitation of generalizability of our findings. Future studies should further verify our conclusions. Fourth, we excluded patients with SUE and SOE in the process of ML because the aim of this study was to identify three main causes of ischemic stroke to bring forward the precise secondary prevention for stroke patients. This step may influence the robustness of the model when applied to the real-world situation. The best situation for this tool is to identify CE, LAA, or SAO when the possibilities of SOE and SUE are low. Fifth, our model inherited some inherent limitations of the TOAST system: (1) SAO was defined by the clinical syndrome and the size of the infarct (≤15 mm in diameter). Consequently, a single larger deep infarct could be classified as SUE rather than a more appropriate diagnosis of SAO. (2) SUE group accounted for approximately 40% of all strokes, including those patients with potential multiple etiologies or patients who had incomplete diagnostic workup.

## Conclusion

In conclusion, the RF model proposed, which combined clinical information, radiologic data, and laboratory testing, could be a useful diagnostic tool to help neurologists quickly give the causative classification of ischemic stroke and initiate the optimal strategies of secondary prevention.

## Data Availability Statement

The raw data supporting the conclusions of this article will be made available by the authors, without undue reservation.

## Ethics Statement

The studies involving human participants were reviewed and approved by the Human Ethics Committee of the Second Affiliated Hospital of Zhejiang University, School of Medicine, and the approval number is yan-2018-102. The patients/participants provided their written informed consent to participate in this study.

## Author Contributions

JW, XG, HC, and ML were involved in the design of the study. JW, WaZ, YC, and YZ did the statistical analysis and wrote the first draft. ML attested that all listed authors meet the authorship criteria. All authors contributed to the further drafts, read and approved the final manuscript.

## Conflict of Interest

The authors declare that the research was conducted in the absence of any commercial or financial relationships that could be construed as a potential conflict of interest.

## Publisher’s Note

All claims expressed in this article are solely those of the authors and do not necessarily represent those of their affiliated organizations, or those of the publisher, the editors and the reviewers. Any product that may be evaluated in this article, or claim that may be made by its manufacturer, is not guaranteed or endorsed by the publisher.
