# Supplementary material for: Causative Classification of Ischemic Stroke by the Machine Learning Algorithm Random Forests
Source: Front Aging Neurosci. 2022 Apr 15;14:788637. doi: 10.3389/fnagi.2022.788637 (PMC9051333; doi:10.3389/fnagi.2022.788637)

**Supplemental Material**

**Data for developing models**

| Demographics and basic information |
| --- |
| Female (0=No, 1=Yes) |
| Age (y) |
| Systolic blood pressure at admission (mmHg) |
| Diastolic blood pressure at admission (mmHg) |
| Body mass index (kg/m^2) |
| Baseline National Institutes of Health Stroke Scale score |
| Baseline modified Rankin Scale score |
| Baseline Glasgow Coma Scale score |
| History of Antiplatelet drug (0=No, 1=Yes) |
| History of anticoagulants (0=No, 1=Yes) |
| History of antihypertensive drugs (0=No, 1=Yes) |
| History of lipid-lowering drugs (0=No, 1=Yes) |
| History of hypoglycemic drugs (0=No, 1=Yes) |
| Smoking (including both currently and previously smoking) (0=No, 1=Yes) |
| Alcohol drinking (including both currently and previously drinking) (0=No, 1=Yes) |
| Previous coronary heart disease (0=No, 1=Yes) |
| Previous atrial fibrillation (0=No, 1=Yes) |
| Previous myocardial infarction (0=No, 1=Yes) |
| Previous valvular heart disease (0=No, 1=Yes) |
| Previous atrial flutter (0=No, 1=Yes) |
| Previous hypertension (0=No, 1=Yes) |
| Previous hyperlipaemia (0=No, 1=Yes) |
| Previous diabetes (0=No, 1=Yes) |
| Previous transient ischemic attack (0=No, 1=Yes) |
| Previous stroke (0=No, 1=Yes) |
| Previous congestive heart failure (0=No, 1=Yes) |
| Previous hyperhomocysteinemia (0=No, 1=Yes) |
| Family history of cardiovascular disease (0=No, 1=Yes) |
| Previous mechanical valve (0=No, 1=Yes) |
| Previous mitral stenosis (0=No, 1=Yes) |
| Previous renal insufficiency (0=No, 1=Yes) |
| Previous malignant tumor (0=No, 1=Yes) |
| Previous systemic lupus erythematosus (0=No, 1=Yes) |
| Previous patent foramen ovale (0=No, 1=Yes) |
| Laboratory information |
| White blood cell count (*10^9/L) |
| Proportion of neutrophils (%) |
| Red blood cell count (*10^9/L) |
| Hemoglobin (g/L) |
| Platelet count (*10^9/L) |
| Prothrombin time (s) |
| International normalized ratio |
| D-dimer (ug/L) |
| Activated partial thromboplastin time (s) |
| Fibrinogen (g/L) |
| Glucose at admission (mmol/L) |
| Homocysteine (umol/L) |
| Total cholesterol (mmol/L) |
| Triglyceride (mmol/L) |
| High-density lipoprotein (mmol/L) |
| Low-density lipoprotein (mmol/L) |
| Image information |
| Both anterior and posterior circulation infarction (0=No, 1=Yes) |
| Anterior circulation infarction (0=No, 1=Yes) |
| Posterior circulation infarction (0=No, 1=Yes) |
| Large vessel occlusion (including unilateral occlusion of intracranial internal carotid artery, or M1/M2 segments of the middle cerebral artery, or basilar artery) (0=No, 1=Yes) |
| Degree of stenosis of intracranial arteries (including intracranial internal carotid artery, or M1/M2 segments of the middle cerebral artery, or basilar artery) [0 =none, 1 =mild (<50%), 2 =moderate (50%-70%), 3 = severe (>70%)] |
| Heart information |
| Newly diagnosed atrial fibrillation (0=No, 1=Yes) |
| Diagnosis at discharge |
| Discharged atrial fibrillation (0=No, 1=Yes) |
| Discharged mitral stenosis (0=No, 1=Yes) |
| Discharged left ventricular hypertrophy (0=No, 1=Yes) |
| Discharged heart failure (0=No, 1=Yes) |
| Discharged coronary heart disease (0=No, 1=Yes) |
| Discharged myocardial infarction (0=No, 1=Yes) |
| Discharged patent foramen ovale (0=No, 1=Yes) |
| Discharged congenital heart disease (0=No, 1=Yes) |
| Discharged hypertension (0=No, 1=Yes) |
| Discharged hyperlipaemia (0=No, 1=Yes) |
| Discharged diabetes mellitus (0=No, 1=Yes) |
| Discharged deep venous thrombosis (0=No, 1=Yes) |
| Discharged transient ischemic attack (0=No, 1=Yes) |
| Discharged atrial flutter (0=No, 1=Yes) |
| Discharged hyperthyroidism (0=No, 1=Yes) |
| Discharged renal insufficiency (0=No, 1=Yes) |
| Discharged congestive heart failure (0=No, 1=Yes) |

**Plots of hyperparameters search**

Plots of Ada Boosting:


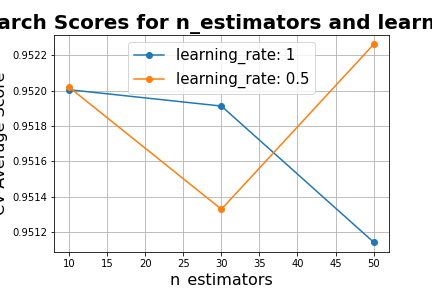


Plots of KNN:


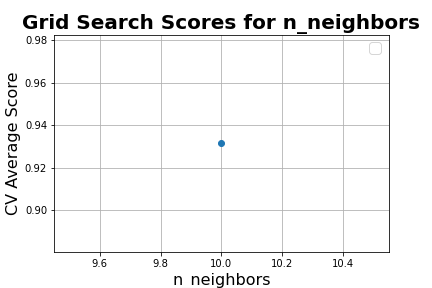

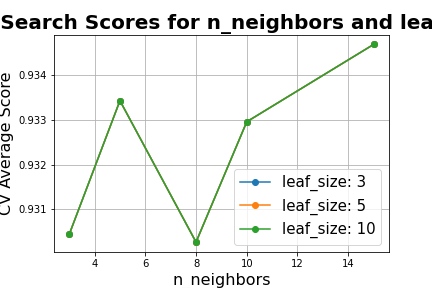

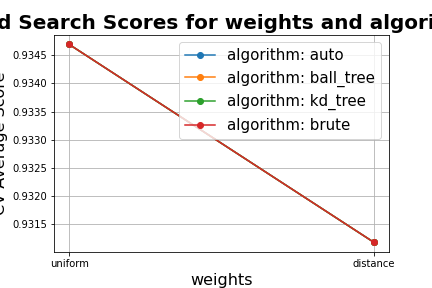


Plots of LR:


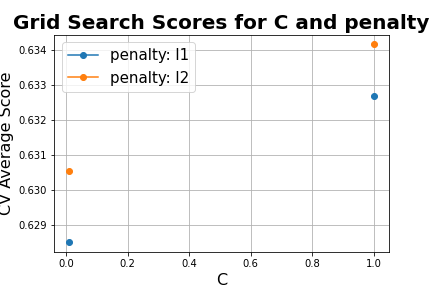

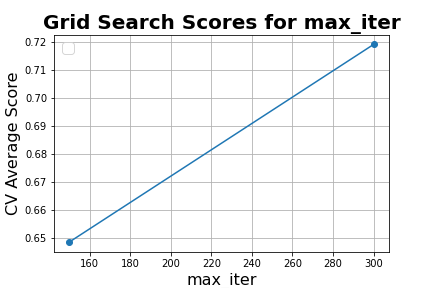


Plots of RF:


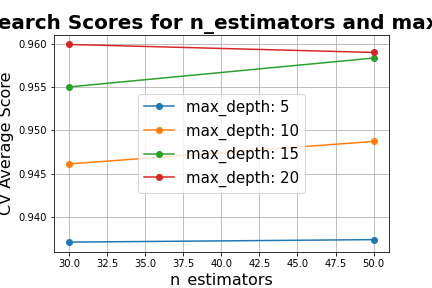

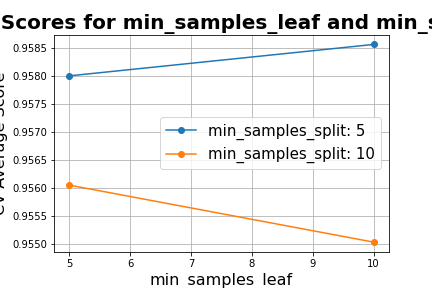

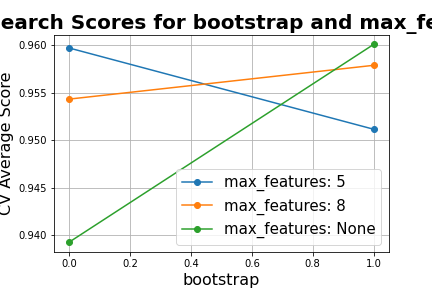

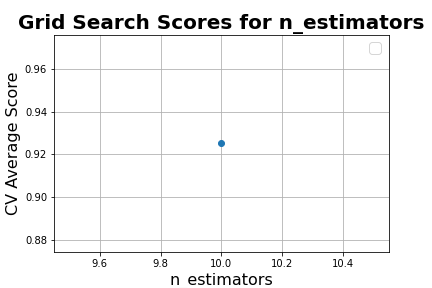


LAA model

Plots of Ada Boosting:


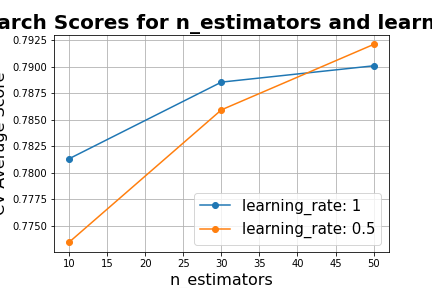


Plots of KNN:


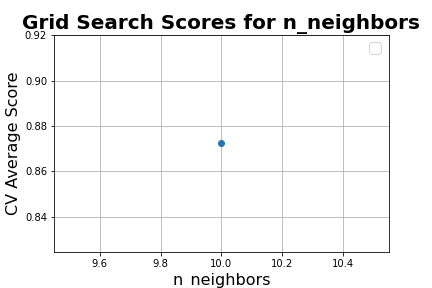

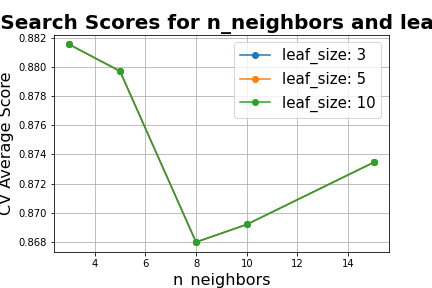

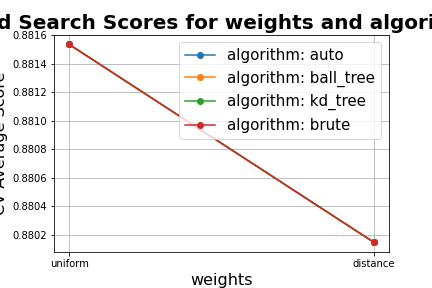


Plots of LR:


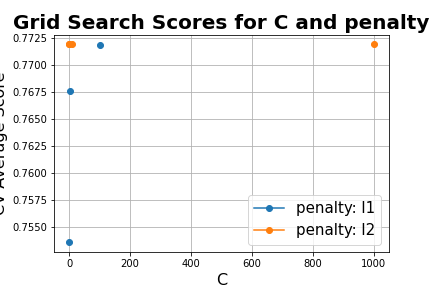

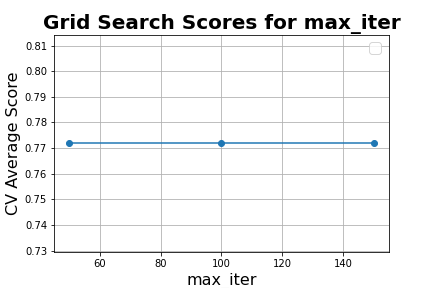


Plots of RF:


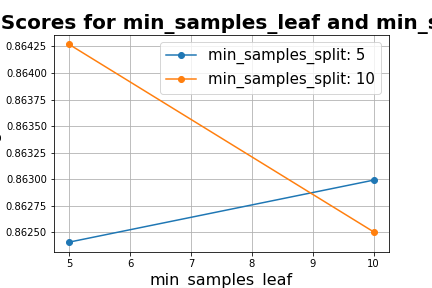

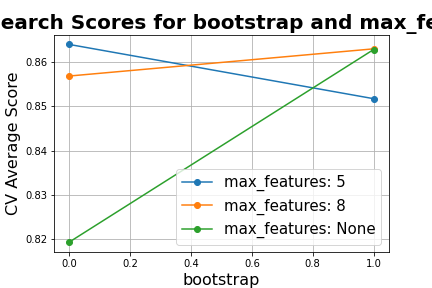

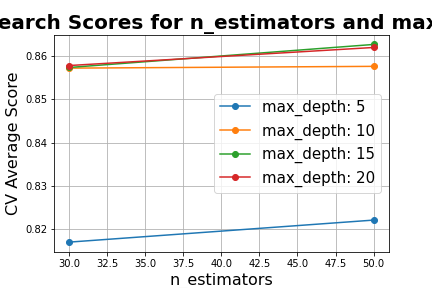


SAO model:

Plots of Ada Boosting:


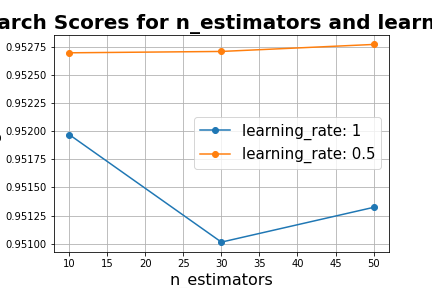


Plots of KNN:


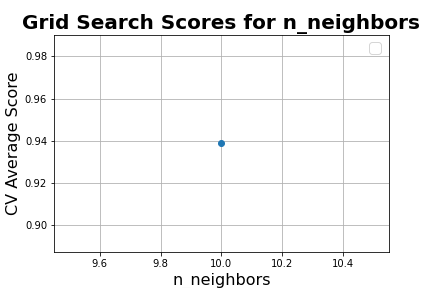

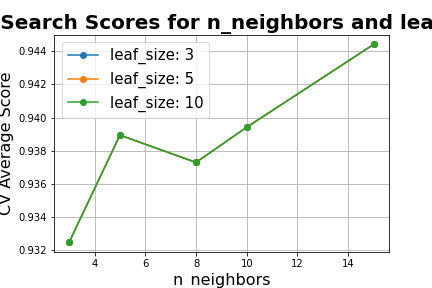

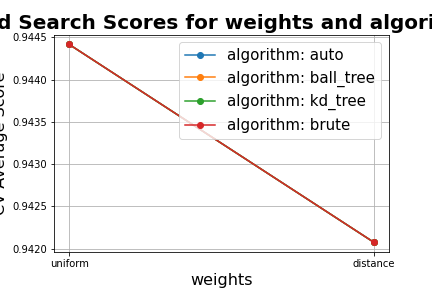


Plots of LR:


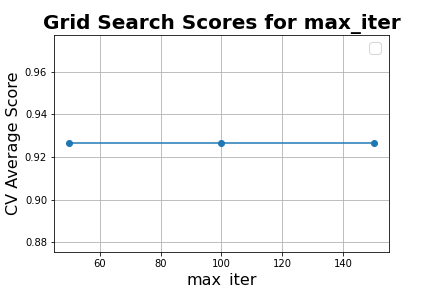

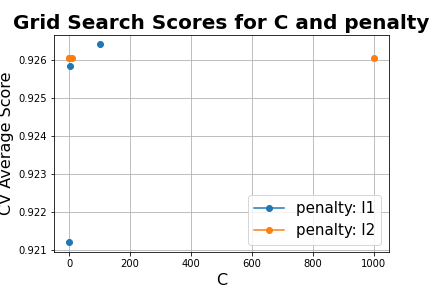


Plots of RF:


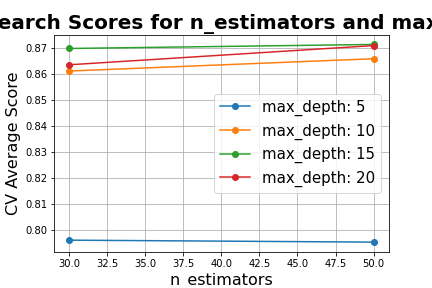

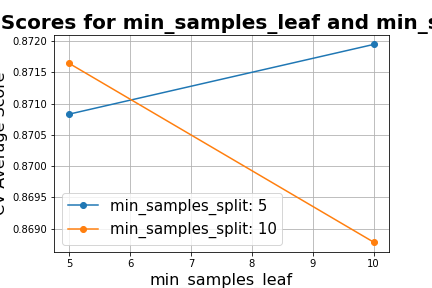

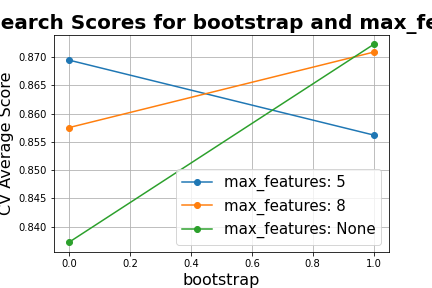

Supplement: Supplementary file 1 [file Data_Sheet_1.docx]
